# High Prevalence of Multimorbidity and Polypharmacy in Elderly Patients With Chronic Pain Receiving Home Care are Associated With Multiple Medication-Related Problems

**DOI:** 10.3389/fphar.2021.686990

**Published:** 2021-06-08

**Authors:** Juliana Schneider, Engi Abd Elhady Algharably, Andrea Budnick, Arlett Wenzel, Dagmar Dräger, Reinhold Kreutz

**Affiliations:** ^1^ Charité - Universitätsmedizin Berlin, Corporate Member of Freie Universität Berlin and Humboldt-Universität zu Berlin, Institute of Clinical Pharmacology and Toxicology, Berlin, Germany; ^2^ Charité - Universitätsmedizin Berlin, Corporate Member of Freie Universität Berlin and Humboldt-Universität zu Berlin, Institute of Medical Sociology and Rehabilitation Sciences, Berlin, Germany

**Keywords:** chronic pain, comorbidity, drug-drug interactions, elderly, medication-related problems, multimorbidity, outpatient, polypharmacy

## Abstract

**Aim:** To measure the extent of polypharmacy, multimorbidity and potential medication-related problems in elderly patients with chronic pain receiving home care.

**Methods:** Data of 355 patients aged ≥65 years affected by chronic pain in home care who were enrolled in the *ACHE* study in Berlin, Germany, were analyzed. History of chronic diseases, diagnoses, medications including self-medication were collected for all patients. Multimorbidity was defined as the presence of ≥2 chronic conditions and levels were classified by the Charlson-Comorbidity-Index. Polypharmacy was defined as the concomitant intake of ≥5 medications. Potentially clinically relevant drug interactions were identified and evaluated; underuse of potentially useful medications as well as overprescription were also assessed.

**Results:** More than half of the patients (55.4%) had moderate to severe comorbidity levels. The median number of prescribed drugs was 9 (range 0–25) and polypharmacy was detected in 89.5% of the patients. Almost half of them (49.3%) were affected by excessive polypharmacy (≥10 prescribed drugs). Polypharmacy and excessive polypharmacy occurred at all levels of comorbidity. We detected 184 potentially relevant drug interactions in 120/353 (34.0%) patients and rated 57 (31.0%) of them as severe. Underprescription of oral anticoagulants was detected in 32.3% of patients with atrial fibrillation whereas potential overprescription of loop diuretics was observed in 15.5% of patients.

**Conclusion:** Multimorbidity and polypharmacy are highly prevalent in elderly outpatients with chronic pain receiving home care. Medication-related problems that could impair safety of drug treatment in this population are resulting from potentially relevant drug interactions, overprescribing as well as underuse.

## Introduction

Over the last few decades, the population of older adult has grown worldwide especially in the developed countries (Mathers et al., 2015). Between 2000 and 2016, the global life expectancy increased by 5.5 years with a mean age of 72 years (World Health Organization, 2019). The number of individuals having two or more chronic conditions, referred to as multimorbidity has also increased with population aging according to a WHO World Health Survey reporting data from 28 countries between 2001 and 2004 (Afshar et al., 2015). The average number of chronic diseases per patient aged over 60 years was estimated to be 5.3 in men and 5.7 in women in Germany (Kostev and Jacob, 2018). Multimorbidity is associated with poorer health outcomes (Xu et al., 2017), higher mortality rates (McPhail, 2016) and impacts profoundly on healthcare utilization and costs (McPhail, 2016).

Polypharmacy is a common clinical consequence of multimorbidity in older adults encompassing not only prescribed but also over-the-counter medications including among others herbal supplements (Pitkälä et al., 2016). Commonly defined as the concomitant use of ≥5 medications daily (Masnoon et al., 2017), polypharmacy is a formidable problem posing a multitude of negative health outcomes (Maher et al., 2014). It increases the risk of adverse drug reactions, adverse drug events (e.g., falls, fractures, and acute kidney injury), inappropriate medication, medication errors, drug-drug interactions (DDI) and increased risk of mortality (Maher et al., 2014; Chang et al., 2020). Moreover, polypharmacy reduces adherence to appropriate pharmacotherapy and may contribute to physical disability and lower cognitive functions (Wastesson et al., 2018). Optimizing prescribing for elderly is of paramount importance as it can improve health outcomes in multimorbid vulnerable patients e.g., patients with chronic pain. Those patients are particularly susceptible to high multimorbidity burdens as well as risk of polypharmacy (Hubbard et al., 2015; Nakad et al., 2020). Moreover, a strong association was found between a high burden of comorbidity and pain severity in elderly (Leong et al., 2007; Blyth et al., 2008). We therefore aimed to analyze the extent of multimorbidity and polypharmacy in elderly chronic pain patients receiving home care and assessed potential medication-related problems in this target group.

## Material and Methods

### Design and Setting

The current analysis is a planned pre-specified subanalysis of the *ACHE* study (“Development of a Model for PAin Management in Older Adults ReCeiving Home CarE”) in Germany. Briefly, *ACHE* was an observational cross-sectional analysis of a population-based cohort of older adults which focused on pain management in home care and has been described previously in detail (Schneider et al., 2020). Ethical approval was obtained by the local ethical committee of the Charité, Universitätsmedizin Berlin (EA1/368/14). Written informed consent was obtained from all participants or their legal guardians in case of cognitive impairment.

### Study Population

Home-dwelling elderly with chronic pain recruited between 09/2017 and 10/2018 in the framework of *ACHE*, aged ≥65 years receiving home care in the city of Berlin, Germany, were eligible for the study (*n* = 355). As cognitively impaired older adults are also at increased risk of multimorbidity and the negative consequences of polypharmacy, patients with cognitive impairment were also eligible for inclusion in *ACHE.* Hence, patients were enrolled independently from their cognitive status as determined by the Mini Mental Status Examination (MMSE) (Tombaugh and McIntyre, 1992).

### Data Collection

**FIGURE 1:**
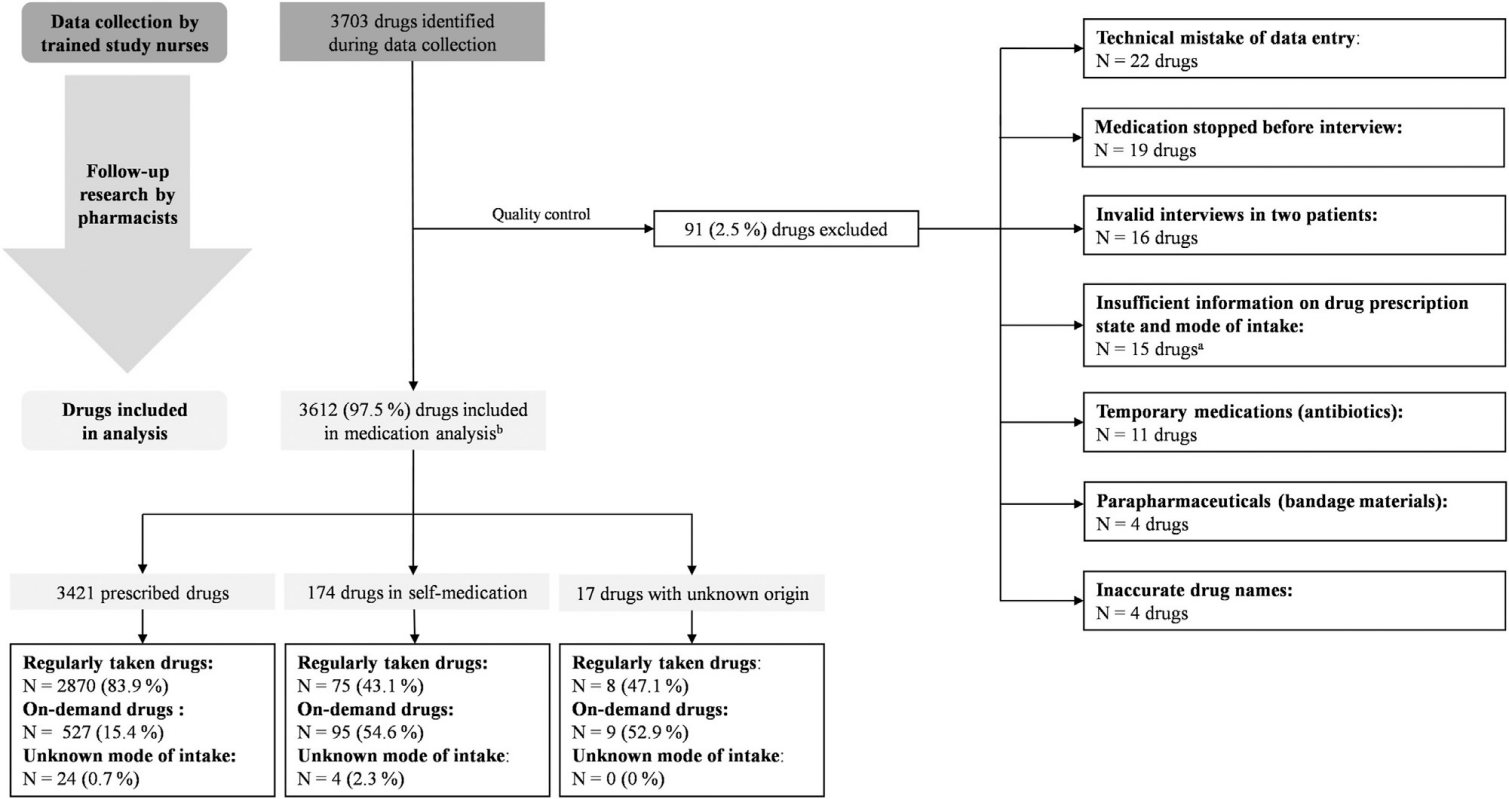
Flow-diagram showing medication screening and selection process. ^a^All drugs were non-prescription drugs. ^b^Different patients may use the same drug.

Five trained investigators interviewed the participants, collected demographic data, the level of care as well as the education level (highest school education and highest professional education) and documented the concurrent medications used regularly or as needed based on drug packages and medication plans available at participants’ homes. According to the Pharmaceutical Care Network Europe (PCNE) classification (Griese-Mammen et al., 2018), we performed an intermediate medication review (PCNE type 2A) based on medication history and patient information. Medications were documented by means of the Instrument for Database-assisted Online recording for Medication (IDOM) (Mühlberger et al., 2003) that based on the data provided by the AOK Research Institute (WIdO). In total, 91 (2.5%) drugs were excluded from medication analysis according to our pre-specified criteria (Figure 1).

For the assessment of comorbidities, history of chronic diseases was obtained by thoroughly reviewing the participants’ medical records, physician reports, nursing records as well as self-reported diagnoses. Polypharmacy was defined as the concomitant intake of ≥5 medications whereas excessive polypharmacy as ≥10 medications, taken regularly or on-demand (Wastesson et al., 2018). Our analysis encompassed all medications including nutraceuticals prescribed by physician(s) or used in self-medication and focused on pharmacologically active ingredients. Drugs were categorized according to the Anatomical Therapeutic Chemical (ATC) classification system. We checked for relevant DDI, over- and underprescriptions in the concurrently prescribed medications within the general context of medication errors defined as events happening during drug treatment and could cause harm to the patient [Pharmacovigilance Risk Assessment Committee (PRAC), 2015]. Regarding the prescription frequency of drug classes and their relevance to our target group, clinically relevant DDI were checked for anticoagulants, diuretics, and statins, namely simvastatin, using our available institutional drug information system AiDKlinik® and their severity was rated on a case-by-case basis by three clinical pharmacologists/pharmacists (JS, EA, RK) according to our pre-defined criteria. In our evaluation of DDI, we also took into account the vulnerable nature of elderly patients and their susceptibility to more risk and more harmful consequences of DDI than that expected in younger patients.

### Instruments and Measures

The individuals’ burden of current diseases in chronic pain patients was assessed by the original Charlson-Comorbidity-Index (CCI) (Charlson et al., 1994) which is a widely used multimorbidity score in older adults (Diederichs et al., 2011). It represents a weighted index of comorbidity based on 19 chronic diseases according to International Classification of Diseases (ICD) diagnosis codes that are weighted differently according to the relative mortality risk (Charlson et al., 1987). We adapted the CCI for use in our population to include also self-reported diagnoses terms when physician reports or nursing records were unavailable. Furthermore, we checked for other chronic diseases such as hypertension, coronary artery disease and atrial fibrillation (AF) besides those listed in the CCI. Multimorbidity was defined as the presence of ≥2 chronic conditions (Kostev and Jacob, 2018). Levels of comorbidity were classified according to the original CCI score as: 0 (no comorbidity), 1–2 (low comorbidity), 3–4 (moderate comorbidity) and ≥5 (severe comorbidity) (Charlson et al., 1987).

We used the CHA_2_DS_2_-VASc [Congestive heart failure/left ventricular dysfunction, Hypertension, Age ≥75 (doubled), Diabetes, Stroke (doubled) – Vascular disease, Age 65–74, and Sex category (female)] score for patients with AF to evaluate their risk of thromboembolism (Hindricks et al., 2021).

### Data Analysis

Descriptive statistics were used to describe patients’ demographics, multimorbidity and medications. Variables were checked for normal distribution by Shapiro-Wilk test and non-parametric tests were used according to the type of variable (continuous or categorical). We used Mann-Whitney U test to check the association of the CCI score (continuous) with sex (categorical), prevalence of polypharmacy (categorical) and excessive polypharmacy (categorical). Chi-squared test was used to examine the association between sex and both polypharmacy and excessive polypharmacy as well as the association of comorbidity levels (categorical) with level of care (categorical) and education level [highest school education (categorical) and highest professional education (categorical)]. Spearman’s correlation was used to test the correlation between the CCI score and the number of prescribed medications (continuous), age (continuous) and MMSE score (continuous). Kruskal-Wallis H test was used for associations between number of prescribed medications (continuous) and levels of comorbidity (categorical with >2 groups). The post-hoc Dunn-Bonferroni test was applied for pairwise comparison between the groups. In addition, we used the Jonckheere-Terpstra test to check for an overall trend between the groups and calculated the corresponding Kendall’s tau-b (τ) correlation coefficient. Data were analyzed using IBM SPSS Statistics, version 25 (IBM Corp, Armonk, NY). Two-tailed statistical significance was assessed at level 0.05.

## Results

### Study Population

A total of 355 patients met the formal inclusion criteria of the *ACHE* study and data were analyzed as two cohorts: the medication-analysis cohort and the multimorbidity cohort (Table 1). For the medication-analysis cohort, data for 353 (99.4%) patients (mean age 82.2 ± 7.5 years, 71.7% females) were available including 22.7% of patients with severe cognitive impairment (MMSE ≤17 points). For the multimorbidity cohort, data of 334 (94.1%) patients (82.2 ± 7.6 years, 71.6% females) were available and 19.1% of them had severe cognitive impairment.

**TABLE 1:** Patients’ characteristics.

Characteristics	Population for medication analysis			Population for multimorbidity analysis		
	Total	Women	Men	Total	Women	Men
	*N* = 353 (99.4%)	*N* = 253 (71.7%)	*N* = 100 (28.3%)	*N* = 334 (94.1%)	*N* = 239 (71.6%)	*N* = 95 (28.4%)
Age (years)	82.2 ± 7.5	83.0 ± 7.1	80.2 ± 8.3	82.2 ± 7.6	82.8 ± 7.1	80.4 ± 8.5
Care level (%)^a^						
1	11.3	9.9	15.0	11.7	10.5	14.7
2	44.8	45.4	43.0	46.1	46.9	44.2
3	21.0	20.2	23.0	21.2	20.1	24.2
4	12.7	13.8	10.0	11.4	12.5	8.4
5	7.4	7.1	8.0	6.9	6.7	7.4
nd	2.8	3.6	1.0	2.7	3.3	1.1
MMSE (%)^b^ ^,^ ^c^						
0–17 points	22.7	23.8	20.0	19.1	20.5	15.8
18–23 points	15.7	15.5	16.0	16.2	15.5	17.9
24–30 points	61.6	60.7	64.0	64.7	64.0	66.3
Number of all drugs^d^ [median, (range)]	10 [0–25]	10 [0–22]	10 [2–25]	10 [0–25]	10 [0–22]	10.5 [2–25]
Number of prescribed drugs^d^ [median, (range)]	9 [0–25]	9 [0–22]	10 [2–25]	10 [0–25]	10 [0–22]	10 [2–25]
Polypharmacy (%)^d^	89.5	89.7	89.0	89.2	89.5	88.3
(≥ 5 prescribed drugs)						
Excessive polypharmacy (%)^d^	49.3	48.2	52.0	51.4	50.2	54.3
(≥ 10 prescribed drugs)						

nd, not determined; MMSE, Mini Mental State Examination.

a According to § 15 SGB XI, the level of care is based on the degree of self-dependence and ranges from 1 (lowest degree) to 5 (most severe impairment with special requirements for nursing care).

b The MMSE-score was calculated for 352/353 of the medication population.

c According to the MMSE classification (Tombaugh and McIntyre, 1992): 0–17 points (severe cognitive impairment), 18–23 points (mild cognitive impairment), 24–30 points (no cognitive impairment).

d Medication data for 333/334 of the multimorbidity population were available.

**FIGURE 2:**
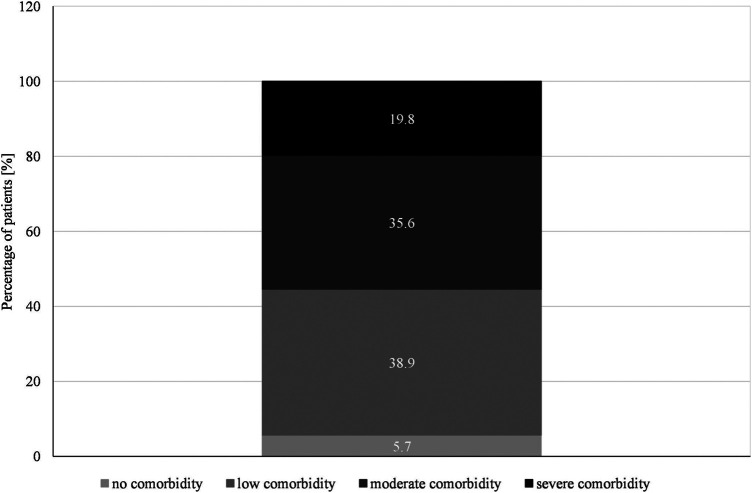
Classification of comorbidities according to the Charlson-Comorbidity-Index (*N* = 334).

The most common diseases found in the multimorbidity cohort were hypertension (78.4%), congestive heart failure (CHF) (41.3%), diabetes with/without organ damage (32.1%), dementia (27.2%), coronary heart diseases (26.9%) and chronic pulmonary diseases (25.1%) (Table 2). Overall, CCI ranged from 0 to 13 with a median score of 3 (IQR: 2–4) in both men and women with more than half of the patients (55.4%) having moderate to severe comorbidity levels (Figure 2). Sex, age, cognitive state, level of care, education level did not significantly affect comorbidity scores. The prevalence of multimorbidity (≥2 chronic diseases) according to the original CCI was 73.7%, and 91.6% when additional disorders detected in the population were counted (Table 2).

**TABLE 2:** Prevalence of comorbidities among elderly receiving home care (*N* = 334).

Comorbid condition	Assigned weights for comorbidities in the CCI	Patients with comorbidity, N (%)^a^
Comorbidities covered by the CCI		
Congestive heart failure	1	138 (41.3)
Dementia	1	91 (27.2)
Chronic pulmonary disease	1	84 (25.1)
Peripheral vascular disease	1	78 (23.4)
Diabetes^b^ with organ damage	2	77 (23.1)
Cerebrovascular disease	1	74 (22.2)
Connective tissue disease	1	50 (15.0)
Myocardial infarction	1	43 (12.9)
Ulcer disease	1	43 (12.9)
Any tumor	2	44 (13.2)
Moderate or severe renal disease	2	39 (11.7)
Diabetes^b^ without organ damage	1	30 (9.0)
Mild liver disease	1	27 (8.1)
Hemiplegia	2	16 (4.8)
Metastatic solid tumor	6	6 (1.8)
Moderate or severe liver disease	3	3 (0.9)
Leukemia	2	2 (0.6)
Lymphoma	2	1 (0.3)
AIDS	6	0 (0)
Additional comorbidities detected in *ACHE*		
Hypertension	–	262 (78.4)
Coronary heart disease	–	90 (26.9)
Atrial fibrillation	–	65 (19.5)
Hemiparesis	–	62 (18.6)
Other arrhythmias	–	49 (14.7)
Prostate disorders	–	35 (10.5)

CCI, Charlson Comorbidity Index.

a Patients may have more than one comorbidity.

b Diabetes includes all patients treated with insulin or oral hypoglycemics, but not diet alone.

### Medication State

**FIGURE 3:**
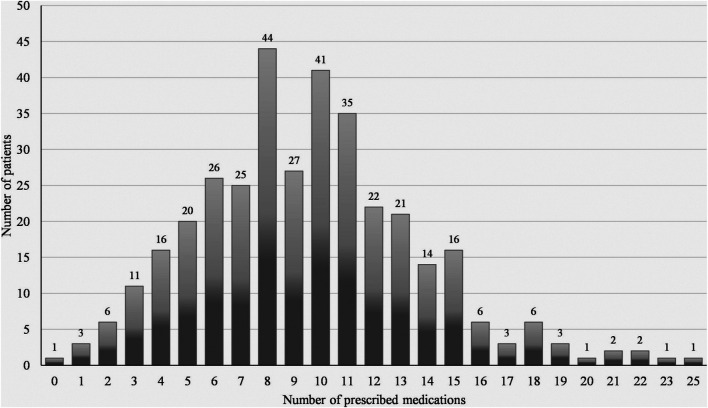
Number of prescribed medications among elderly receiving home care (*N* = 353).

**FIGURE 4:**
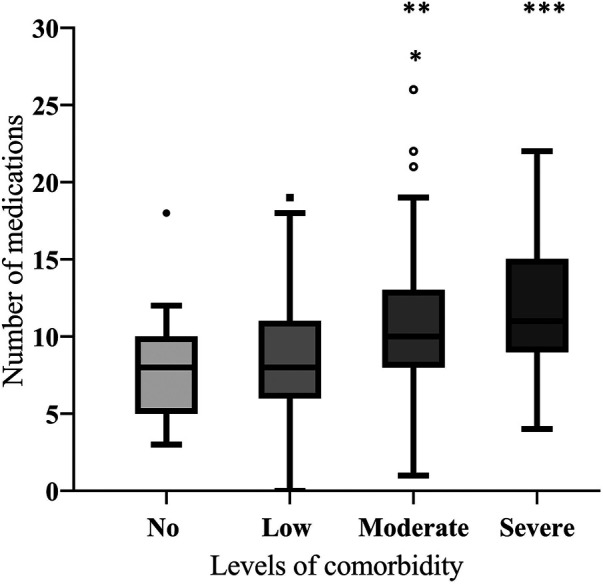
Boxplot diagram: Number of prescribed drugs grouped by different comorbidity levels.*p < 0.05 vs. no comorbidity; **p < 0.01 vs. low comorbidity; ***p < 0.001 vs. low comorbidity; ***p < 0.001 vs. no comorbidity.

Among the medication-analysis cohort, 3,703 medication products were screened during data collection yielding 3,612 (97.5%) medication products for analysis after screening for data quality as per our preset criteria (Figure 1). Of those, 3,421 (94.7%) medication products were prescribed by physicians and 174 (4.8%) were used in self-medication, while for 17 (0.5%) medication products, the prescription mode could not be verified. According to the ATC code, analgesics, diuretics, antithrombotics, renin-angiotensin system (RAS) blockers were most frequently prescribed [Supplementary Table S1 of the Electronic Supplementary Material (ESM)]. The median number of prescribed drugs was 9 (range 0–25), and 10 (range 0–25) when self-medication was accounted for (Table 1). A highly significant positive correlation was found between the CCI and the number of prescribed drugs (r_s_ = 0.345, *p* < 0.001). The prevalence of polypharmacy (≥5 prescribed drugs) was 89.5% (*n* = 316) and almost half of the patients (*n* = 174; 49.3%) were affected by excessive polypharmacy (≥10 prescribed drugs) (Figure 3). There were no sex-specific differences for the prevalence of either polypharmacy or excessive polypharmacy (Chi-squared test, *p* = 0.842, and *p* = 0.522, respectively). Patients affected by prescribed polypharmacy had significantly higher CCI scores (median: 3, range 0–13) than patients without polypharmacy (median: 2, range 0–5, Mann Whitney test, U = 3077.5, *p* < 0.001). Similarly, excessive polypharmacy was also associated with higher CCI scores (median: 4, range 0–13, Mann-Whitney test, U = 9271.5, *p* < 0.001). Moreover, significant associations were found between the number of prescribed medications and different levels of comorbidity (Kruskal-Wallis test, H = 36.3, *p* < 0.001). The adjusted *p*-values of the post-hoc analysis are shown in Figure 4. In addition, we found an overall positive trend between these groups (τ = 0.262, *p* < 0.001). Polypharmacy and excessive polypharmacy were detected in all levels of comorbidity.

### Medication Errors Detected in Selected Drugs/Drug Classes (Anticoagulants, Diuretics, and Simvastatin)

In total, 184 clinically relevant potential DDI from which 57 (31.0%) evaluated as severe were detected in more than a third (34.0%) of patients in the medication-analysis cohort (Supplementary Table S2 of the ESM). DDI lacking clear clinical meaning or consequences were not presented. Over- and underprescription of drugs were detected.

#### Anticoagulants

##### Drug-Drug Interactions

A total of 80 patients received anticoagulants in the medication analysis cohort for which 27 potential and 12 severe interactions were detected in 28 (35.0%) patients (Supplementary Table S2 of the ESM).

##### Underprescription (Subgroup Analysis for AF)

In our multimorbidity cohort, 65/334 (19.5%) patients (mean age 82.8 ± 7.4 years) had AF (Table 2). All of them achieved a CHA_2_DS_2_-VAS score ≥2 (median: 5; range 3–8) but only 44 (67.7%) patients were anticoagulated with direct oral anticoagulants (DOAC) or vitamin-K-antagonist (VKA), while 21 (32.3%) received no oral anticoagulants; of these, 19 patients were ≥75 years old. The three most prescribed anticoagulants were apixaban (36.4%), phenprocoumon (29.5%) and rivaroxaban (20.5%).

#### Diuretics

##### Drug-Drug Interactions

Among the diuretics, all diuretic agents including potassium-sparing diuretics were included in DDI evaluation amounting to 281 diuretic prescriptions in 224 patients. We found 131 potential DDI, 36 (27.5%) of them were evaluated as severe (Supplementary Table S2 of the ESM).

##### Overprescription (Subgroup Analysis for Loop Diuretics)

A total of 195/334 (59.9%) patients were treated with diuretics. By far, the most commonly prescribed diuretic was torasemide, prescribed in 160 (82.1%) patients. Loop diuretics were combined with thiazide/thiazide-like diuretics in 11/195 (5.6%) patients. In one patient, torasemide and furosemide were even co-prescribed. Among 174 (89.2%) patients receiving loop diuretics, 27 (15.5%) patients had no documented indication for CHF, advanced chronic kidney disease or edema.

#### Simvastatin

Overall, 85/353 (24.1%) patients took simvastatin once daily in an average dose of 29.9 ± 14.5 mg. We found 17 potential DDI between simvastatin and other drugs (e.g., amlodipine, dronedarone, colchicine, ranolazine). Of these, 11 interactions were rated as severe (Supplementary Table S2 of the ESM).

#### Demonstration of Patient Case Study Affected by Excessive Polypharmacy and DDI

**FIGURE 5:**
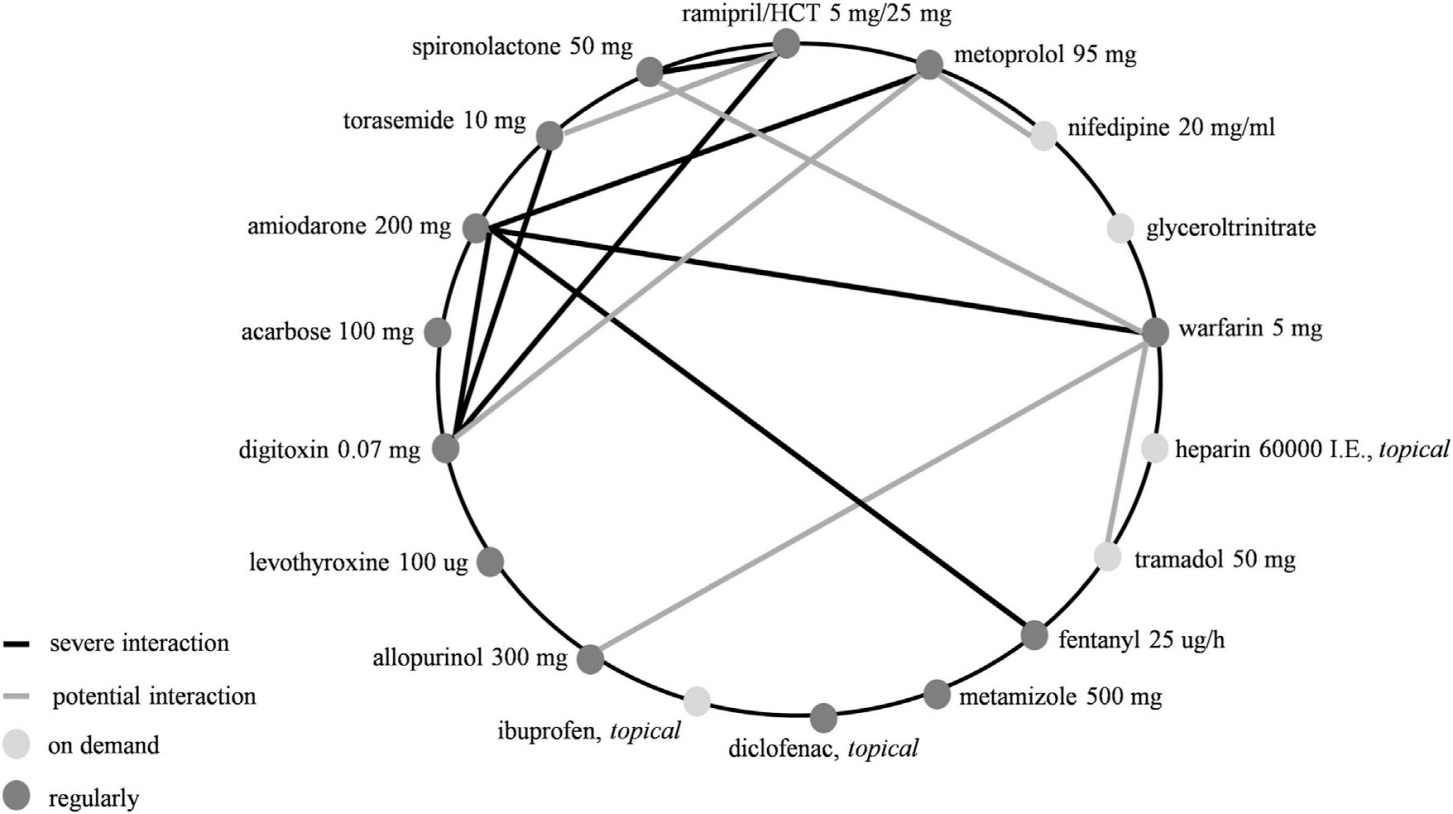
Drug-drug interactions of prescribed medication in one patient.

One patient (83 years, female) for whom we checked the whole DDI profile appears of interest (Figure 5). The patient had following comorbidities: hypertension, CHF, AF, diabetes with organ damage, connective tissue disease, edema, and hemiparesis. She was prescribed 18 different drugs by the physician. The patient had suffered a stroke in the past, had a CHA_2_DS_2_-VASc score of 8 and a CCI score of 5. We identified 13 potential clinically relevant interactions. Of these, seven could be severe (Supplementary Table S2 and Figure 5).

## Discussion

The present study revealed that multimorbidity and polypharmacy along with the consequences of polypharmacy (e.g., higher risk of medication errors, DDI, inappropriate medication use) were highly prevalent in our cohort of older adults with chronic pain receiving home care that also included patients with severe cognitive impairment. To the best of our knowledge, this is the first cross-sectional prospective study in Germany to examine the burden of multimorbidity and polypharmacy in this setting involving chronic pain patients. Chronic pain has been reported to be associated with high burden of comorbid diseases and high risk of polypharmacy (Fishbain, 2005; Paladini et al., 2015; Jokanovic et al., 2016). In one study, chronic pain was independently associated with higher daily drug consumption (Ersoy and Engin, 2018).

We found a median CCI score of 3 (IQR: 2–4) in both men and women and an overall range of 0–13. More than half of the patients (55.4%) had moderate (35.6%) to severe (19.8%) comorbidity levels (Figure 2). However, these values could be still underestimated considering patients for whom morbidity scores could not be determined due to lack of self-report or medical records. In addition, all patients, by virtue of our study design, had chronic pain. CCI scores ≥3 have been correlated with an increased risk of hospital readmission (Halfon et al., 2002), while scores ≥5 correlated significantly with mortality and high risk of medication errors (Charlson et al., 1987; Rabenberg et al., 2019). The KORA-Age study (mean age 73.4 ± 6.1 years) reported a median number of conditions of 2 (IQR: 1–3) and a multimorbidity (≥2 chronic conditions) prevalence of 58.6% (95% CI: 57.0-60.2) (Kirchberger et al., 2012). Our higher multimorbidity rate (91.6%) could be ascribed to the home care setting and the older age of our patients. For the assessment of comorbidities, Kirchberger et al. (Kirchberger et al., 2012) also used a CCI generated self-reported diagnoses and included hypertension, eye diseases, mental and neurological diseases, that were deemed highly relevant for exploring multimorbidity in the elderly (Kirchberger et al., 2012). Bravo and colleagues also extended the Charlson list of 19 comorbidities to include 10 other disorders being significant to mortality or functional decline in long-term care setting such as valvular heart diseases (Bravo et al., 2002). Despite the limitation of the CCI to detect other relevant disorders, the CCI is still widely used to investigate comorbidity in geriatric patients in healthcare research (Jorgensen et al., 2012; Abizanda et al., 2014; Rochon et al., 2014; Gellert et al., 2019).

The median number of prescribed drugs was 9 (range 0–25) and 10 (range 0–25) when self-medication was included. In nursing homes in Germany, an average of 5.9 ± 3 (range 0–16) drugs prescribed concomitantly per resident was reported (Kolzsch et al., 2012), while in general practice, a mean of 4.2 ± 2.7 in men and women aged ≥60 years, about 37% of whom were affected by polypharmacy (≥5 prescribed drugs) (Kostev and Jacob, 2018). We found a higher prevalence of polypharmacy (89.5%) with almost half of the patients (49.3%) having excessive polypharmacy (≥10 prescribed drugs) in our study, which may relate to the high multimorbidity prevalence in our study as a driver for polypharmacy. The mean number of prescribed medications was significantly associated with higher CCI-based morbidity levels supporting the reciprocal link between multimorbidity and polypharmacy. The latter acts as a driver for medication-related morbidity and increases the chance of DDI to which elderly patients are more vulnerable. In our target group, potential DDI were detected in a third of patients.

DDI involving simvastatin, a well-known substrate of cytochrome P450 (CYP) 3A4 (Tiwari et al., 2006) predisposing to myotoxicity were detected. The risk of myotoxicity is elevated with older age as muscle mass decreases (Kellick et al., 2014), with renal impairment, and high dose therapy (Tiwari et al., 2006).

As a case study, we demonstrated the overall DDI profile of a multimorbid female patient affected by excessive polypharmacy and experiencing a complex drug regimen. For this patient, we detected several DDI involving anticoagulants and diuretics. This case also illustrates an increased number of prescribed drugs proportional to a high CCI, with a comorbidity score of 5. Notably, guideline-based treatments for several diseases facilitate polypharmacy as illustrated in this patient treated for CHF, hypertension and AF and was therefore included in the AF subgroup analysis.

Patients with AF and a CHA_2_DS_2_-VASc score ≥2 should be anticoagulated with DOAC or VKA due to risk of stroke (Hindricks et al., 2021). In our study, 19.5% of patients had AF with a median CHA_2_DS_2_-VASc score of 5 (range 3–8). However, about a third (32.3%) of them did not receive anticoagulant therapy with either DOAC or VKA suggesting a state of underprescription of potentially useful medications. Though current AF management guidelines recommend oral anticoagulant treatment at age ≥75 years regardless of additional risk factors for stroke (Hindricks et al., 2021), underuse of oral anticoagulant treatment in the elderly with AF has been previously reported (Zarraga and Kron, 2013; Steinberg et al., 2015; Kreutz et al., 2018).

Diuretics, commonly prescribed in the elderly, often cause hypovolemia and hyponatremia which increase the risk of falling that was associated with higher morbidity and mortality in older adults (Maher et al., 2014). Elderly hypertensive patients were more likely to develop hyponatremia after age 75 years (Diaconu et al., 2014). Loop diuretics were prescribed in 15.5% of patients without a documented appropriate indication. This includes edematous disorders due to CHF, hepatic cirrhosis or nephrotic syndrome, and advanced renal insufficiency (Sarafidis et al., 2010). Additionally, 5.6% of patients on diuretics received concomitantly loop diuretics and thiazide/thiazide-like diuretics. Overprescription of loop diuretics without appropriate indication has been reported in 27.5% of nursing home residents (Kölzsch et al., 2010). The concomitant use of spironolactone and ramipril as illustrated in our patient case increases the risk of hyperkalemia; a potentially severe DDI to which the elderly are more sensitive due to potassium homeostasis abnormalities, disorders e.g., diabetes mellitus or use of drugs e.g., RAS blockers and potassium-sparing diuretics (Hunter and Bailey, 2019).

Suboptimal prescribing in elderly includes, besides unnecessary prescribing or overprescribing, underuse or underprescribing of indicated medications (Devik et al., 2018). The latter is defined as failure to prescribe a potentially useful drug and has become a frequent problem leading to adverse clinical consequences e.g., stroke in high risk patients undertreated for atrial fibrillation (Kuijpers et al., 2008). Polypharmacy can also be a driver for medication underuse reported to occur in over 40% of patients with polypharmacy (Kuijpers et al., 2008).

This study is the first to examine the burden of multimorbidity and polypharmacy in older adults with chronic pain receiving home care. The strengths of our study lie in the rigorous evaluation of the drug profile including self-medication and drugs prescribed regularly or on-demand as well as including patients with severe cognitive impairment. In contrast to previous studies that excluded patients with cognitive impairment (Markotic et al., 2013; Nawai et al., 2017), patients with cognitive impairment were eligible for inclusion in *ACHE*. However, the following limitations are acknowledged:

First, this is a cross-sectional cohort study. As such, patients were interviewed once; follow-up data of patients were not available. In addition, contacts with the treating physician were not implemented in the study design. Hence, it was not possible to assess the persistence of polypharmacy or notify the physician in case of suspected DDI or trace the outcome of the potential DDI whether a corrective action was taken by the physician or a follow-up for clinical condition was undertaken. Second, our sample size was small, and the study reflects local data to the city of Berlin regarding patients with chronic pain in the home care setting which may limit the generalizability of our findings concerning prevalence rates of multimorbidity and polypharmacy and its consequences. Nevertheless, we preferred to analyze qualitatively the prescribed medications rather than to systematically report the prevalence of medication errors, DDI and inappropriate medication use to get an insight into the consequences of polypharmacy in multimorbid chronic pain patients. This highlights also how significant DDI could be regardless of their actual prevalence and helps instigate awareness on the harmful effects of DDI in this group.

## Conclusion

Multimorbidity and polypharmacy are highly prevalent in elderly outpatients with chronic pain receiving home care. Regular monitoring and evaluation of medications in this population appears thus important together with strategies aiming to optimize therapy by addressing differential aspects of medication-related problems including drug interactions, overprescribing as well as underuse.

## Data Availability

The datasets presented in this article are not readily available because data are archived in the Institute of Clinical Pharmacology and Toxicology, Charité – Universitätsmedizin Berlin, and can be accessed by all interested researchers on site. Requests to access the datasets should be directed to RK, reinhold.kreutz@charite.de.
