# Supplementary material for: High Prevalence of Multimorbidity and Polypharmacy in Elderly Patients With Chronic Pain Receiving Home Care are Associated With Multiple Medication-Related Problems
Source: Front Pharmacol. 2021 Jun 8;12:686990. doi: 10.3389/fphar.2021.686990 (PMC8217758; doi:10.3389/fphar.2021.686990)
Supplement: Supplementary file 1 [file DataSheet1.PDF]

Electronic Supplementary Material

SUPPLEMENTARY TABLE 1 | Prescribed drug classes according to ATC code in elderly receiving home care (N = 353).

	Drug class	ATC code	Drugs, N (%)
**1**	Analgesics	N02	406 (11.24 %)
**2**	Diuretics	C03	250 (6.92 %)
**3**	Antithrombotics	B01	232 (6.42 %)
**4**	Renin-angiotensin system blockers	C09	231 (6.40 %)
**5**	Drugs for acid related disorders	A02	200 (5.54 %)
**6**	Antidiabetics	A10	189 (5.23 %)
**7**	Beta blockers	C07	181 (5.01 %)
**8**	Lipid lowering agents	C10	141 (3.90 %)
**9**	Vitamins	A11	138 (3.82 %)
**10**	Laxatives	A06	137 (3.79 %)
**11**	Psychoanaleptics	N06	130 (3.60 %)
**12**	Drugs for obstructive airway diseases	R03	113 (3.13 %)
**13**	Calcium channel blockers	C08	108 (2.99 %)
**14**	Psycholeptics	N05	100 (2.77 %)
**15**	Antiepileptics	N03	96 (2.66 %)
**16**	Antianaemics	B03	91 (2.52 %)
**17**	Thyroid therapy	H03	91 (2.52 %)
**18**	Topical drugs for muscle and joint pain	M02	75 (2.08 %)
**19**	Non-steroidal anti-inflammatory drugs	M01	66 (1.83 %)
**20**	Ophthalmics	S01	65 (1.80 %)
**21**	Cardiac therapy	C01	50 (1.38 %)
**22**	Dietary supplements	V06	50 (1.38 %)
**23**	Antigout preparations	M04	48 (1.33 %)
**24**	Drugs for functional gastrointestinal disorders	A03	40 (1.11 %)
**25**	Mineral supplements	A12	40 (1.11 %)
**26**	Antiparkinsonism drugs	N04	40 (1.11 %)
**27**	Urological drugs	G04	39 (1.08 %)
**28**	Systemic Corticosteroids	H02	28 (0.78 %)
**29**	Antihypertensives	C02	24 (0.66 %)
**30**	Drugs for bone diseases	M05	24 (0.66 %)
**31**	Others[a]	-	189 (5.23 %)
	Total	-	3612 (100 %)

[a]Drugs accounting for less than 20 prescriptions.

SUPPLEMENTARY TABLE 2 | List of clinically relevant drug-drug interactions in *ACHE* (N = 353) with their potential risks and recommended interventions.

**Interacting drugs**[a]	**Number of patients affected by the interaction**	**Potential risks**	**Mechanism**	**Management**	**Severe DDI**
**Anticoagulants**					
Rivaroxaban + citalopram (SSRI) or duloxetine (SNRI)	2	Citalopram or duloxetine may potentiate the risk of bleeding in patients treated with anticoagulants.	SSRIs and SNRIs alter platelet function and may induce bleeding because serotonin released by platelets plays an important role in hemostasis.	Monitor for clinical and laboratory signs for hematologic complications; consider prophylactic treatment with proton pump inhibitors to reduce gastrointestinal bleeding risk; consider use of other antidepressant drugs e.g., mirtazapine, trazodone, bupropion.	
Rivaroxaban + clopidogrel	1	Combination of two antithrombotic drugs (anticoagulant and platelet inhibitor) increases bleeding risk.	Added effect of two antithrombotic drugs.	The benefit-risk for each individual patient should be assessed, monitor carefully for bleeding events.	
Phenprocoumon + omeprazole or esomeprazole	3	Increased anticoagulant activity of phenprocoumon. INR increases were reported, and dose reduction was required in individual cases.	Omeprazole and esomeprazole may reduce the clearance of phenprocoumon due to competitive inhibition of its degradation.	Monitor INR with concomitant use and after discontinuation of omeprazole or esomeprazole.	

Phenprocoumon + allopurinol	3	Increased phenprocoumon plasma level and possible increase in INR doubling the risk of bleeding.	Allopurinol reduces the elimination of phenprocoumon by inhibiting its metabolism.	Monitor INR with concomitant use and after discontinuation of allopurinol.	
Phenprocoumon + sertraline, citalopram or escitalopram (SSRI)	4	SSRIs may potentiate the risk of bleeding in patients treated with anticoagulants.	SSRIs alter platelet function and may induce bleeding because serotonin released by platelets plays an important role in hemostasis.	Monitor for clinical and laboratory signs for hematologic complication; consider prophylactic treatment with proton pump inhibitors to reduce gastrointestinal bleeding risk; consider use of other antidepressant drugs e.g., mirtazapine, trazodone, bupropion.	
Phenprocoumon + metformin	3	Metformin reduces the AUC of phenprocoumon up to 63 % and increases dose requirements.	Unclear; metformin may increase phenprocoumon clearance by either enhancing liver perfusion or bile salt excretion resulting in a faster elimination of phenprocoumon.	Monitor INR with concomitant use and after discontinuation of metformin; adjust carefully phenprocoumon dosage.	
Phenprocoumon + mirtazapine	1	Prolongation of clotting time and increased risk of bleeding.	Mirtazapine can decrease the metabolism of phenprocoumon.	Monitor INR carefully; switch to another antidepressant if necessary.	
Warfarin + spironolactone	1	Spironolactone may attenuate anticoagulation effects of warfarin especially with high doses.	Unclear.	Monitor INR with concomitant use and after discontinuation of spironolactone.	

Warfarin + allopurinol	1	Prolongation of warfarin half-life and enhancement of its anticoagulant effect.	Allopurinol may inhibit the metabolism of warfarin.	Monitor INR with concomitant use and after discontinuation of allopurinol.	
Warfarin + tramadol	1	Increase in INR and risk of bleeding; more frequent in carriers of the slow CYP2D6 allele.	Unclear; possibly related to reduced activity of CYP2D6 by tramadol.	Monitor INR with concomitant use and after discontinuation of tramadol; dose reduction of warfarin to 70 % may be necessary.	
Enoxaparin + ASA[b]	1	Combination of two antithrombotic drugs (anticoagulant and platelet inhibitor) increases bleeding risk.	Added effect of two antithrombotic drugs.	Use cautiously only when necessary, with close clinical and laboratory observation for bleeding complications and neurological impairments.	✓
Apixaban, rivaroxaban or edoxaban + ibuprofen[c]	5	Increased risk of bleeding.	NSAIDs inhibit platelets and increased gastrointestinal bleeding risk and can increase exposure to anticoagulants eliminated by renal clearance by acute kidney injury.	Avoid use of NSAIDs in elderly patients with high risk of bleeding; reduce other modifiable bleeding risk factors.	
Apixaban or rivaroxaban + ASA[b]	2	Combination of two antithrombotic drugs (anticoagulant and platelet inhibitor) increases bleeding risk.	Added effect of two antithrombotic drugs.	Use cautiously only when necessary, with close clinical and laboratory observation for bleeding complications and neurological impairments.	✓

Rivaroxaban + oxcarbazepine	1	Oxcarbazepine decreases the plasma concentrations of rivaroxaban and increases the risk of therapeutic failure and thrombosis.	Oxcarbazepine induces CYP3A4 for which rivaroxaban is a substrate.	Avoid use; consider alternative antithrombotic drug (e.g., warfarin).	✓
Phenprocoumon + L-thyroxine	4	L-thyroxine may increase the risk of bleeding at initiation of therapy.	L-thyroxine increases the metabolism of vitamin K-dependent coagulation factors; not specific for phenprocoumon but all anticoagulants including with similar mechanism of action.	Titrate the dose slowly; monitor INR or prothrombin time when initiating, discontinuing, or changing the dosage of L-thyroxine in stabilized patients and adjust dose accordingly.	✓
Phenprocoumon + ibuprofen[c]	1	Increased risk of bleeding.	NSAIDs inhibit platelets and increased gastrointestinal bleeding risk and can increase exposure to anticoagulants eliminated by renal clearance by acute kidney injury.	Avoid use of NSAIDs in elderly patients with high risk of bleeding; reduce other modifiable bleeding risk factors.	
Phenprocoumon + ASA[b]	1	Increased risk of bleeding.	Added effect of two antithrombotic drugs.	Use cautiously only when necessary, with close clinical and laboratory observation for bleeding complications and neurological impairments.	✓

Warfarin + amiodarone	1	Amiodarone may increase the pharmacologic effects of warfarin.	Amiodarone inhibits CYP2C9 and CYP3A4 hepatic metabolism of S-warfarin, poor CYP2C9 metabolizers may have a higher risk of bleeding and a faster onset of the interaction.	Reduce anticoagulant dosage by 30 % to 50 %; monitor within first 7 weeks of therapy prothrombin time or INR; monitor for signs of excessive anticoagulation.	✓
**Diuretics**					
Loop diuretics					
Furosemide or torasemide + ACEi[d]	67	Hyponatremia and hypovolemia increase the risk of falling for elderly patients who are more prone to develop hyponatremia after age 75 years. ACEi may cause renal insufficiency or acute renal failure in patients with sodium depletion.	Co-administration increases risk of hypotension and hypovolemia than does either drug alone especially with high doses of loop diuretics.	Monitor blood pressure, renal function, and electrolytes during co-administration.	
Furosemide or torasemide + digitalis glycosides	8	Increased risk for digitalis-induced arrhythmias.	Diuretic-induced hypokalemia and hypomagnesemia.	Monitor for potassium and magnesium levels; check for signs of possible digoxin toxicity or electrolyte disturbances; adjust digitalis dose.	✓

Thiazide diuretics/thiazide-like diuretics					
HCT, xipamide or indapamide + digitalis glycosides	4	Increased risk for digitalis-induced arrhythmias.	Diuretic-induced hypokalemia, hypomagnesemia and hypercalcemia.	Monitor potassium, magnesium and calcium levels; check for signs of possible digoxin toxicity or electrolyte disturbances; adjust digitalis dose.	✓
HCT, xipamide or indapamide + ACEi[d]	28	Hyponatremia and hypovolemia increase the risk of falling for elderly patients who are more prone to develop hyponatremia after age 75 years. ACEi may cause renal insufficiency or acute renal failure in patients with sodium depletion.	Thiazide-induced water and sodium depletion increase the risk of hypotension and hypovolemia when co-administered with ACEi especially with high doses of thiazide diuretics.	Monitor blood pressure, renal function and electrolytes during co-administration.	
Potassium-sparing diuretics					
Spironolactone, eplerenone, triamterene or amiloride + RAS blockers	24	Increased the risk of hyperkalemia in patients with risk factors such as renal impairment, diabetes, old age, severe or worsening heart failure, and concomitant use of potassium supplements.	RAS blockers increase serum potassium which could be additive with that induced by potassium-sparing diuretics.	Use with caution; monitor closely for signs of hyperkalemia; check renal function regularly; avoid potassium supplementation.	✓
**Statins**					
Simvastatin (30, 40 mg) + amlodipine	4	Higher risk of statin-induced myopathy.	Amlodipine inhibits simvastatin metabolism via intestinal and hepatic	Simvastatin dosage should not exceed 20 mg daily when	✓

			CYP3A4 resulting in higher plasma concentrations of simvastatin and its active metabolite simvastatin acid.	used in combination with amlodipine.	
Simvastatin (20 mg) + amlodipine	4			Avoid co-administration at the same time; separate dosing by at least 4 h.	
Simvastatin + dabigatran	2	Simvastatin increases the risk of bleeding associated with the use of dabigatran by 44 %.	Via P-gp inhibition of simvastatin and increase in dabigatran-etexilate absorption.	Avoid co-administration; switch to another statin other than simvastatin or lovastatin.	✓
Simvastatin + carbamazepine	1	Carbamazepine decreases AUC of simvastatin by 25 % and its active metabolite simvastatin acid by 18 %.	Carbamazepine induces the metabolism of simvastatin and simvastatin-acid via intestinal and hepatic CYP3A4.	Increase the dosage of simvastatin, switch to other statins such as pravastatin metabolized by non-CYP routes, and rosuvastatin with lower metabolic fraction.	✓
Simvastatin + warfarin	1	Simvastatin enhances the anticoagulant response to warfarin depending on the CYP2C9 genotype, reduces dosage requirement of warfarin by 29 % in carriers of the slow metabolizing allele (CYP2C9*3) and by 43 % in homozygous carriers.	Possibly due to selective inhibition of CYP2C9*3 allele by simvastatin.	Closer monitoring of INR, switch to another statin not affecting anticoagulation such as pravastatin.	
Simvastatin + phenytoin	1	Phenytoin reduces the lipid lowering efficacy of simvastatin.	Phenytoin induces CYP enzymes including CYP3A4 isoenzyme thus increasing the metabolism of simvastatin.	Adjust dose of simvastatin; monitor cholesterol levels especially with familial hypercholesterolemia due to higher cardiovascular risk.	✓

Simvastatin + sacubitril /valsartan	1	Sacubitril may increase the plasma concentrations of simvastatin increasing the risk of statin-induced myopathy.	Sacubitril inhibits hepatic uptake transporters OATP1B1 and/or OATP1B3 to which statin are substrate.	Monitor for signs of myopathy or rhabdomyolysis.	
Simvastatin + dronedarone	1	Dronedarone increases the plasma concentrations of simvastatin and metabolite simvastatin acid increasing the risk of statin-induced myopathy.	Dronedarone inhibits both intestinal P-gp and hepatic/intestinal CYP3A4 isoenzyme enhancing absorption as well as reducing simvastatin and simvastatin acid clearance.	Adjust dose; monitor for signs of myopathy or rhabdomyolysis; switch to other non P-gp/CYP3A4 substrates e.g., fluvastatin, pitavastatin, or rosuvastatin.	✓
Simvastatin + colchicine	1	Increased risk of myopathy and rhabdomyolysis by pharmacodynamic and pharmacokinetic interactions.	Both drugs are myotoxic and may have additive or synergistic effects when used together. Both are P-gp/CYP3A4 substrates, competitive inhibition may occur resulting in increased drug absorption and decreased excretion.	Monitor for signs of myopathy or rhabdomyolysis; monitor creatine kinase level after co-administration and after any dose increase.	✓
Simvastatin + ranolazine	1	Ranolazine increases the plasma concentrations of simvastatin and simvastatin-acid increasing the risk of statin-induced myopathy.	Ranolazine inhibits intestinal and hepatic CYP3A4.	Simvastatin dosage should not exceed 20 mg daily; switch to other non P-gp/CYP3A4 substrates e.g., fluvastatin, pitavastatin, or rosuvastatin.	✓

Further clinically relevant interactions demonstrated in one patient					
Amiodarone + metoprolol	-	Additive effects of severe bradycardia, hypotension, or cardiac arrest.	Amiodarone increases the AUC of metoprolol by 80 %. The extent of increased AUC depends on CYP2D6-genotype.	Avoid this combination or monitor blood pressure and ECG.	✓
Amiodarone + fentanyl	-	Increased risk for cardio-depressive effects.	Coadministration with inhibitors of CYP3A4 such as amiodarone may increase the plasma concentrations of fentanyl, which is metabolized by this isoenzyme.	Monitor for signs of fentanyl toxicity (e.g., dizziness, confusion, fainting, extreme sedation, bradycardia, shortness of breath); adjust dosage if necessary.	✓
Amiodarone + digitoxin	-	Increased risk for digitalis toxicity.	Coadministration with amiodarone may increase serum digoxin concentrations by up to 100 %, frequently resulting in clinic[a]l toxicity.	Adjust dosage; monitor serum digitalis level and observe patients for clinical evidence of digitalis toxicity (e.g., nausea, anorexia, visual disturbances, slow pulse, irregular heartbeats).	✓
Metoprolol + nifedipine, *fast-acting* (on-demand)	-	Increased hypotension and risk of heart failure.	Additive cardiovascular effects.	Monitor blood pressure; use prolonged release preparations for regular use.	
Metoprolol + digitoxin	-	Increased risk for bradycardia.	Concomitant use of digitalis glycosides and beta-blockers may increase the risk of bradycardia and AV-block.	Monitor heart rate, ECG for AV block.	

DDI, drug-drug interaction; SSRI, selective serotonin reuptake inhibitor; SNRI, serotonin noradrenaline reuptake inhibitor; INR; International Normalized Ratio; AUC, area under the concentration-time curve; CYP, cytochrome P450; ASA, acetyl salicylic acid; NSAID, nonsteroidal anti-inflammatory drug; ACEi; angiotensin converting enzyme inhibitors; HCT, Hydrochlorothiazide; RAS blockers, renin angiotensin system blockers

including ACEi and angiotensin receptor blockers (ARB); P-gp, P-glycoprotein; OATP, organic anion transporting polypeptides; ECG, electrocardiogram, AV block; atrioventricular block.

[a]For the drug interaction analysis, all active ingredients in mono- and combination -preparations were considered. Some patients in *ACHE* were treated with more than one active ingredient belonging to the same drug class.

[b]ASA in 100 mg dose.

[c]Ibuprofen (400 - 600 mg) taken on-demand.

[b]This interaction applies also to ARB.
